# Don’t Let Me Do That! – Models of Precommitment

**DOI:** 10.3389/fnins.2012.00138

**Published:** 2012-10-08

**Authors:** Zeb Kurth-Nelson, A. David Redish

**Affiliations:** ^1^Wellcome Trust Centre for Neuroimaging, University College LondonLondon, UK; ^2^Department of Neuroscience, University of MinnesotaMinneapolis, MN, USA

**Keywords:** discounting function, decision-making, neuroeconomics, temporal diference reinforcement learning, precommitment

## Abstract

Precommitment, or taking away a future choice from oneself, is a mechanism for overcoming impulsivity. Here we review recent work suggesting that precommitment can be best explained through a distributed decision-making system with multiple discounting rates. This model makes specific predictions about precommitment behavior and is especially interesting in light of the emerging multiple-systems view of decision-making, in which functional systems with distinct neural substrates use different computational strategies to optimize decisions. Given the growing consensus that impulsivity constitutes a common point of breakdown in decision-making processes, with common neural and computational mechanisms across multiple psychiatric disorders, it is useful to translate precommitment into the common language of temporal difference reinforcement learning that unites many of these behavioral and neural data.

It seems illogical on the surface, but humans and other animals sometimes put themselves in situations to prevent themselves from being given an option that they would choose if given the chance. They will even expend effort and cost to avoid being given the future option. Such restriction of one’s own future choices is called **precommitment**. It is theorized that precommitment occurs because humans and other animals have different preferences at different times (Strotz, 1955; Ainslie, 1992). Precommitment behaviors take many forms, ranging from purely external mechanisms like flushing cigarettes down the toilet, to purely internal mechanisms like making a promise to oneself that one is unwilling to break, to intermediate mechanisms like making a public statement about one’s intentions.

Precommitment is ubiquitous in human behavior. “Christmas Clubs,” popularized during the Great Depression, enforced saving through the year for Christmas shopping (Strotz, 1955). In the modern era, websites like stickk.com automatically transfer money from a credit card to a designated recipient (such as a charity) if the user fails to meet a specified goal (as reported by a trusted third party). In Australia, Canada, and Norway, many gambling machines require the gambler to pre-set a limit on his or her expenditure, after which the machine deactivates (Ladouceur et al., 2012). (Some gamblers also spontaneously create their own precommitment strategies, Wohl et al., 2008; Ladouceur et al., 2012.) In day-to-day experience, people place the ice cream out of sight, put money into a retirement account with withdrawal penalties, walk a different route to avoid seeing a store where there is temptation to buy something, or self-impose deadlines with self-imposed punishments (Ariely and Wertenbroch, 2002).

Precommitment behavior has been demonstrated in animals (Rachlin and Green, 1972; Ainslie, 1974), but there is not yet an established laboratory paradigm for eliciting precommitment behavior in humans. Although precommitment can be predicted to occur as a direct consequence of time-dependent changes in preference order (Ainslie, 1992), explicit neural and computational models of precommitment remain limited. In our paper, “A reinforcement learning model of precommitment in decision-making” (Kurth-Nelson and Redish, 2010), we examined whether current computational models of decision-making can explain precommitment and what those models imply for the mechanisms that underlie precommitment. Here, we will focus on integrating those results into the broader picture of decision-making.

## Valuation and Discounting

Psychologists and economists (and now, neuroeconomists) operationalize the decision-making process through the framework of *valuation*. Whenever an organism (which we will call an “agent” here, to allow for easy translation between simulations and real organisms) is faced with a choice, each possible outcome is assigned a value. These values are compared, and the outcomes with higher values are more likely to be chosen (Glimcher, 2008). Although there are additional action-selection systems which do not work this way (such as reflexes), there is a compelling body of evidence that valuation plays a role in the making of many choices. Neural correlates of value-based decision-making have been identified in many parts of the brain (Rangel et al., 2008; Kable and Glimcher, 2009).

Rewards become less valued as they are more delayed – a phenomenon known as *temporal* or **delay discounting**. A *discounting function* is a quantitative description of this decay in value (Ainslie, 1992; Mazur, 1997; Madden and Bickel, 2010). The discounting function of an individual human subject can be measured empirically with a series of questions (for example, “Would you prefer $30 today or $100 in a year?”), and is generally stable over time (Ohmura et al., 2006; Takahashi et al., 2007; Jimura et al., 2011).

**Figure 1:**
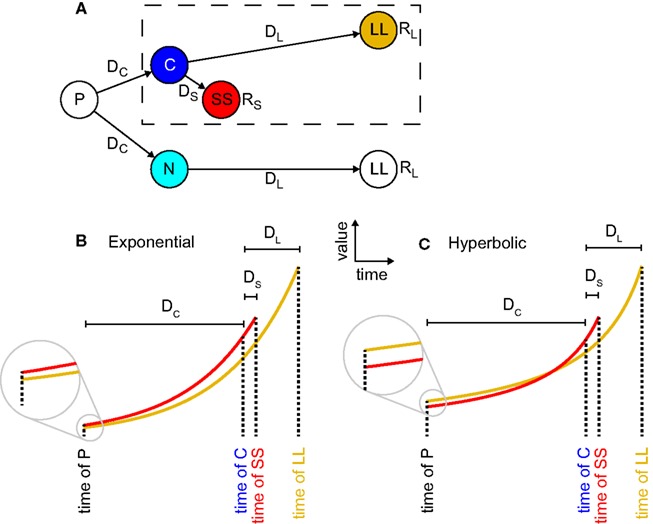
**Precommitment arises from hyperbolic but not exponential discounting**. **(A)** A state-space for precommitment (from Kurth-Nelson and Redish, 2010). The agent first chooses whether to enter state C or state N. From state C, a standard intertemporal choice is available, between a larger reward available later (LL) and a smaller reward available sooner (SS). This choice is outlined with a dashed box. But from state N, only LL is available. Thus choosing N represents precommitment. **(B)** In exponential discounting, values decay by the same percentage for each unit of delay, so if SS is preferred at state C, it must also be preferred at state N. **(C)**, In hyperbolic discounting, values decay more steeply proximally to the outcome, so it is possible for SS to be preferred at state C, but for LL to be preferred at state N.

The simplest discounting function is one that decays exponentially. In *exponential* discounting, each unit of delay reduces value by the same percentage. However, when measured empirically, the discounting functions of humans and animals are not exponential (Ainslie, 1992; Madden and Bickel, 2010). Instead, they are steeper than exponential at short delays, and shallower than exponential at long delays (Figure 1). Hyperbolic functions are often used to fit these curves, but for our purposes it is not critical whether the shape is actually hyperbolic; only that it is more concave than exponential. All non-exponential functions show **preference reversals** (Strotz, 1955; Frederick et al., 2002) – an option preferred today is not necessarily preferred tomorrow.

Precommitment can be explained as a consequence of preference reversal (Ainslie, 1992; Kurzban, 2010).; (Figure 1). Fundamentally, precommitment entails both the preference *at one time* for a smaller reward available sooner (smaller-sooner, SS) over a larger reward that one must wait for (larger-later, LL) – and also the preference *at an earlier time* for LL over SS. In the diagram of Figure 1A, an agent with a preference reversal would prefer SS over LL in situation C, but LL over SS in situation P. This means that in situation P the agent has an incentive to prevent itself from reaching choice C, and to instead go to situation N, in which it has no choice – thereby *precommitting* to LL.

## Computational Models of Precommitment

**Temporal difference reinforcement learning (TDRL)** is often used to bridge the gap between descriptive theoretical models of decision-making and their neural implementation. Because of its biological plausibility, guaranteed convergence, and power to explain behavior and neural activity (Schultz et al., 1997; Sutton and Barto, 1998; Roesch et al., 2012), TDRL has become a well-established model of value-based decision-making (Montague et al., 1996; Schultz, 1998).

TDRL assumes that an agent can take actions, some of which are rewarded. The goal is to learn to take actions that maximize the reward received (Sutton and Barto, 1998). Distinct situations of the world are represented as *states*. TDRL aims to estimate the *value* of each state, which is defined as the total discounted future reward expected from that state. This is a recursive definition: the value of a state can be defined as the discounted value of the next state plus the reward available in the next state (Bellman, 1957). To learn these values, on every state transition, TDRL calculates the difference between the discounted value of the new state (plus the reward received if any) and the value of the old state. This difference defines a prediction error in the value estimation. When this prediction error, scaled by a learning rate, is added to the value estimate, the estimated value is brought closer to the true value. Under appropriate conditions (a stable world, complete exploration, etc.), the value function will converge to the true value function, and, once the values associated with each state are learned, optimal behavior can be achieved by selecting the available action leading to the highest-value state. Although the basic model of TDRL is incomplete (Niv et al., 2006; O’Doherty, 2012; van der Meer et al., 2012), it remains the starting point for computational models of decision-making.

In the standard implementation of TDRL, there is a state transition on every time step. Exponential discounting can therefore be calculated very straightforwardly by taking the value of the current state to be the value of the next state (plus the reward received if any) times a constant γ (0 < γ < 1). In this formulation, each unit of time causes the same attenuation of value, which is the definition of exponential discounting. However, non-exponential discounting has been difficult to implement in TDRL. There have been a handful of attempts at performing non-exponential (specifically, hyperbolic) discounting within a TDRL model (Daw, 2000, 2003; Kurth-Nelson and Redish, 2009; Alexander and Brown, 2010). We examined precommitment behavior in these four TDRL models (Kurth-Nelson and Redish, 2010).

**Figure 2:**
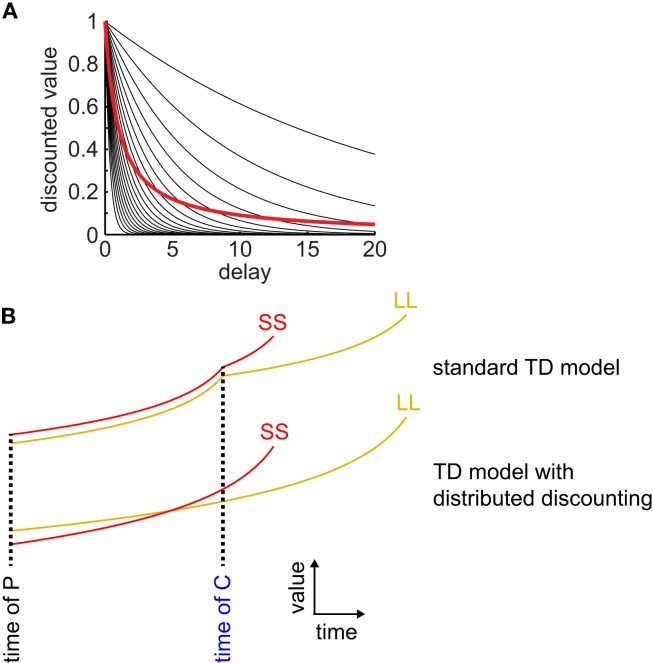
**Distributed discounting enables precommitment in temporal difference learning**. **(A)** Twenty exponential curves with discounting rates spread uniformly between 0 and 1 are shown in black. The average of these curves is shown in red. This average curve closely approximates a hyperbolic function. **(B)** Standard TD models cannot precommit because, at each state transition, discounting starts over, ensuring that if SS is preferred over LL at the time of C, then it is also preferred at the time of P (*top pair of curves*). When averaging a set of exponential discount curves, discounting is not reset at each state transition, so preferences can reverse between C and P (*bottom pair of curves*).

We found that three of these four models produced hyperbolic discounting only in special cases (either across a single state transition, or in an environment with no choices) and therefore were unable to produce precommitment. The other model produced hyperbolic discounting in arbitrary state-spaces and was able to produce precommitment. The successfully precommitting model was the μAgents model that we introduced in 2009 – in this model, a set of exponentially discounting TDRL agents operating in parallel, each with a different discounting rate, and each maintaining its own estimate of the value function, collectively approximate hyperbolic discounting behavior (Figure 2; Kurth-Nelson and Redish, 2009). By using a distributed representation of value, the μAgents model can track hyperbolic discounting across multiple state transitions. The distributed representation of value used by the μAgents model can represent more than just the mean expected value of a given state. This allows the μAgents model to discount hyperbolically across multiple state transitions, which enables preference reversal and therefore precommitment.

A TDRL model of precommitment gives us a concrete computational hypothesis with which to explore potential mechanisms by which people choose to precommit. More generally, it is also important to have computational models that describe choice in complex state-spaces (Kurth-Nelson and Redish, 2012). For example, the same model that allows the analysis of precommitment can also be used to analyze *bundling*. Bundling is another strategy that may be used to overcome an impulsive discounting function (Ainslie and Monterosso, 2003), but unlike precommitment, bundling does not require advance preparation. In bundling, choices are treated as categorical. For example, rather than thinking “Do I want to smoke one cigarette?” one would think, “Do I want to smoke cigarettes?” Non-exponential discounters will often say yes to the former question and no to the latter, at the same time (Rick and Lowenstein, 2008). This dichotomy suggests a multi-faceted value function, such that different components of the valuation process lead to different answers and internal conflict which needs to be resolved before an action can be taken (Kurzban, 2010; van der Meer et al., 2012; Wunderlich et al., 2012).

## Predictions about Precommitment Behavior

Computational models allow exploration of parameter spaces. Although the fact that non-exponential (e.g., hyperbolic) discounting leads to preference reversals (Strotz, 1955; Frederick et al., 2002) and to the potential for precommitment (Ainslie, 1992, 2001) has been noted for decades, several non-intuitive consequences appeared when precommitment was implemented in a computational model.

First, the theoretical model predicts that precommitment is increased when there is a larger contrast between the SS and LL options. In other words, precommitment will be more favored if LL is very large and very delayed, compared to SS (of course, if LL is very large but not very delayed, then it will simply be preferred over SS at any time point, and precommitment will not be required). This suggests that, in the case of addiction, if we want to encourage precommitment, it is important to define the perceived alternative to drug use as being a major outcome, such as the long-term health and safety of oneself or family members (Heyman, 2009). It is less likely that people would spontaneously precommit if the only perceived alternative to drug use were a modest outcome such as saving the money one would have spent on the drugs. Recent work on contingency management (CM, in which a concrete alternative is offered to remain abstinent from drugs) suggests that the most effective CM procedures entail working toward a very large concrete reward far in the future (such as a big-screen television; Petry, 2012).

Second, we can predict that there is a complex effect of an agent’s discounting rate on their ability to precommit. When an agent is highly impulsive (fast discounting rate), it will be highly sensitive to the delay between precommitment and choice. If this delay is small, precommitment is unfavorable, but as this delay increases, the preference for precommitment increases steeply. On the other hand, if an agent is relatively patient (slow-discounting rate), then it will be largely insensitive to the delay between precommitment and choice, exhibiting at best a mild preference for precommitment for any value of this delay. Thus, the highest overall preference for precommitment appears in the most impulsive agents. On the surface this appears a bit paradoxical: the people with the strongest preference for an impulsive choice are the ones most likely to employ a strategy that curtails their ability to reach it. However, this finding suggests that in addiction, treatment strategies should be tailored to the individual depending on his or her own discounting rate. For fast discounters, inserting more time between precommitment and choice is essential – while for slow discounters, the theory predicts that it won’t make much of a difference. In fact, for slow-discounting addicts, precommitment may not be a useful strategy at all.

**Figure 3:**
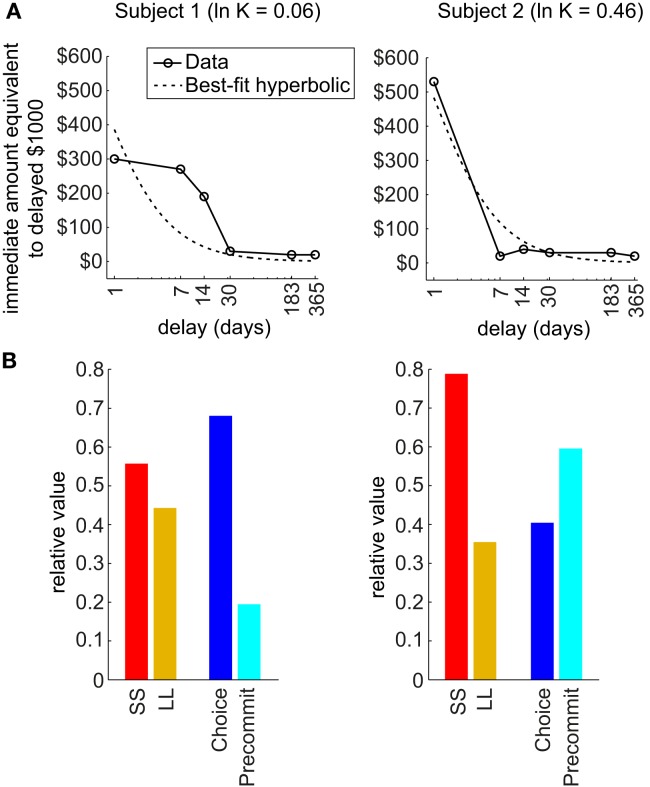
**Shape of discounting curve strongly influences precommitment**. **(A)** The actual discounting curves of two individuals are shown in solid lines, and the best-fit hyperbolic curves are shown in dashed lines. These two subjects were both fit by a hyperbolic function with ln(*K*) of approximately 0 (from a range of −13 to +4 across subjects). **(B)** Predicted precommitment behavior, based on actual discounting curve shape of each subject, using the following parameters: *D*_C_ = 6 days, *D*_L_ = 1 day, *D*_S_ = 0, *R*_L_ = $150, *R*_S_ = $100. Subject 1 is expected to have a modest preference for SS over LL, and to be averse to precommitment. Meanwhile, subject 2 is expected to have a strong preference for SS over LL, but to favor precommitment. (Data from Chopra et al., 2009, used with permission.)

Third, the model predicts that precommitment is highly sensitive to the precise *shape* of an agent’s discounting function (Figure 3). Our theoretical analysis reveals that two discounting functions that are both fit by nearly identical hyperbolic parameters can exhibit entirely different patterns of precommitment behavior. In particular, the simulations in Kurth-Nelson and Redish (2010) illustrated that precommitment depends on the shape of the tail of the discounting function. When the tail of the discounting function is slightly depressed, precommitment behavior can be abolished for some ranges of reward magnitudes and delays. This finding indicates that beyond tailoring treatment to an individual’s best-fit discounting rate, it may provide further therapeutic power to design behavioral interventions most likely to work with the shape of the individual’s discounting function. Additionally, any treatments that modulate the shape of the discounting function may produce large effects on precommitment behavior. For example, boosting serotonin appears to preferentially select slow-discounting components (Tanaka et al., 2007; Schweighofer et al., 2008), which should boost the tail of the discounting curve and improve precommitment.

## Multiple Systems

As noted above, TDRL models are incomplete descriptions of the full range of animal (including human) behavior (O’Doherty, 2012). Recent work suggests that there are at least three behavioral controllers functioning in tandem: habitual, deliberative, and Pavlovian (Daw et al., 2005, 2011; Dayan et al., 2006; Redish et al., 2008; Fermin et al., 2010; Glascher et al., 2010; Simon and Daw, 2011; Huys et al., 2012; van der Meer et al., 2012; Wunderlich et al., 2012). This leads to the **multiple-systems theory of decision-making**, which says that multiple decision-making controllers interact to make decisions. Habitual decision-making entails incremental learning of inflexible stimulus-action relationships that are released upon exposure to certain stimuli; deliberative decision-making entails search and evaluation through a representation of the causal structure of the world; and Pavlovian decision-making entails the release of species-specific approach and avoidance reactions in response to unconditioned or conditioned stimuli. TDRL is generally taken to be a model of habitual behavior.

There are two basic possibilities for how preference reversals, and therefore precommitment, arise within the context of these multiple systems. The first possibility is that preference reversals are inherent within a single instrumental system. For example, precommitment may arise entirely within the habitual system as a consequence of multiple exponential discount rates operating in parallel. In this case, precommitment would exist even without an interaction between multiple systems, and would occur without conscious anticipation of a preference reversal; it would occur entirely as a consequence of differential reinforcement (Ainslie, 1974).

The second possibility is that preference reversals stem from interactions between systems (Bechara et al., 1998; McClure et al., 2004; Dayan et al., 2006; Haidt, 2006; Kurzban, 2010). For example, the deliberative system may discount exponentially, such that LL is preferred from C within the deliberative system; but when faced with an imminent choice of SS, the Pavlovian system adds to the total value of SS such that it is ultimately chosen. From the vantage of P, SS is not imminent, so the Pavlovian approach is absent and the deliberative system can choose N without hindrance. Thus there is an apparent reversal of preference. Reversal could also arise from an interaction between the habitual and deliberative systems. Suppose that the habitual system has a faster discounting rate than the deliberative system and that it dominates at state C (where there is less uncertainty, Daw et al., 2005). Meanwhile, the deliberative system, with a slower discounting rate, dominates at state P. The transition from deliberative to habitual control between P and C would lead to an observed preference reversal.

In other words, the deliberative system would have insight into the expected future impulsive choice of the habitual system, and would choose to take an action leading to a situation where the habitual or Pavlovian system would not have the impulsive action available. Interestingly, explicit insight or cognitive recognition of future **impulsivity** is sometimes assumed to be necessary for precommitment (Baumeister et al., 1994; Kurzban, 2010; Baumeister and Tierney, 2011), but the extent to which precommitment depends on insight is unknown at this time.

These two possibilities suggest different ways in which our model of precommitment (Kurth-Nelson and Redish, 2010) fits into the broader context of multiple decision-making systems. In the first case, the TDRL model describes precommitment within the instrumental habit learning system and is agnostic to the interaction of this system with other decision-making systems. In the second case, the model illustrates the general principle that multiple simultaneous processes with different effective discounting rates produce precommitment. These processes may be a mixture of goal-directed and habitual systems, or a mixture of instrumental and Pavlovian systems. In the second case, the model’s prediction that precommitment is sensitive to the exact shape of the effective discount curve implies that precommitment is sensitive to the exact interplay between systems. Particularly intriguing is the role of the deliberative system in shaping precommitment. The deliberative system entails searching through future possibilities, which suggests that decisions are strongly influenced by the cognitive process of search, and by the representations of those possibilities (Kurth-Nelson et al., 2012). If this deliberative role in precommitment is in fact the case, then precommitment is likely to have a complex interaction with cognitive processes like working memory.

## Computational Psychiatry

Psychiatry is the study of dysfunction within cognitive and decision-making systems. Whereas traditional psychiatry classifies dysfunctions into categories based on external similarities, new proposals have suggested that classification would be better served by addressing the underlying dysfunction. The emerging field of computational psychiatry suggests that computational models of underlying neural mechanisms can provide a more reasoned basis for the nature of dysfunction and the modality of treatment (Redish et al., 2008; Maia and Frank, 2011; Montague et al., 2012).

Impulsivity is a strong candidate for such a trans-disease mechanism (Bickel et al., 2012; Robbins et al., 2012). Impulsive choices underlie several different psychiatric disorders (American Psychiatric Association, 2000; Heyman, 2009; Madden and Bickel, 2010), and there appear to be similar neural bases for impulsivity across these disorders (Dalley et al., 2008; Robbins et al., 2012).

Precommitment is a powerful strategy to combat impulsivity. Although addicts have faster discounting rates on average than non-addicts (Bickel and Marsch, 2001), the distributions of addicts’ and non-addicts’ discounting rates overlap substantially. Furthermore, of all major psychiatric disorders, addiction has by far the highest rate of spontaneous remission (Heyman, 2009), despite the stability of discounting rates over time (Kirby, 2009). This suggests that people can overcome addiction despite continuing to have impulsive underlying preferences. Precommitment is an ideal strategy for an impulsive agent to make healthy choices. Some people may spontaneously acquire precommitment strategies, while others may benefit from being explicitly instructed in such strategies.

Models of precommitment (Kurth-Nelson and Redish, 2010) make predictions about what precommitment strategies will be most effective in treating impulsivity disorders such as addiction. They predict that more impulsive individuals will be more sensitive to the delay between the option to precommit and the availability of the impulsive choice. They also predict that precommitment depends on the precise shape of the discounting curve, such that two individuals with the same discounting rate can exhibit very different precommitment behavior.

The latter is particularly interesting in light of the fact that it is possible to change an individual’s discounting function. For example, Bickel et al. (2012) found that working memory training decreases impulsivity. Others have shown that differences in executive function abilities predict differences in impulsivity (Burks et al., 2009; Romer et al., 2011), which suggests that improving executive function could reduce impulsivity. On the other hand, imposing cognitive load makes subjects more impulsive (Vohs and Faber, 2007; Vohs et al., 2008). It is not yet known whether the improvements in discounting functions from working memory training are due to strengthening of long-sighted neural systems, weakening of short-sighted neural systems, or a change in the interplay between the two. Nor is it yet known how these manipulations interact with precommitment as a treatment paradigm for addiction.

Finally, the TDRL model depends on having a state-space where precommitment is available as an option. This opens the very important and poorly explored question of how the brain constructs the state-space. In the context of the issues examined here, the brain needs to recognize that precommitment is available. It may be that factors such as working memory and other cognitive resources are important for flexibly constructing adaptive state-spaces, and this may be an essential part of recovery. Even verbally instructing an individual that precommitment is available might be enough to help create the state-space that TDRL or other learning processes could use for precommitment. The ability to form representations of the world that support healthy strategies, even in the face of high underlying impulsivity, may be one of the most important factors in recovery from disorders like addiction.

## Conflict of Interest Statement

The authors declare that the research was conducted in the absence of any commercial or financial relationships that could be construed as a potential conflict of interest.

## Key Concept

Precommitment: Taking away a choice from one’s future self in order to enforce one’s present preferences.
Delay discounting: The attenuation in subjective value of rewards that will be delivered in the future. Delay discounting is typically measured by posing decisions between smaller immediate rewards and larger delayed rewards. Subjects with steep discounting will demand a large increase in the magnitude of a reward in order to tolerate a delay in its receipt.
Preference reversal: An instability in preferences over time, such that at one time, X is preferred over Y, but at another time, Y is preferred over X. Preference reversal is central to impulsivity disorders. For example, drugs are rarely preferred over healthy choices when the choice is viewed from a distance, but often preferred when immediately available. Therefore it is critical to have mechanisms to enforce the healthy preferences.
Temporal difference reinforcement learning (TDRL): A standard computational framework that helps to explain behavioral and neural data. TDRL works by calculating a prediction error at each time step, which encodes the difference between expected and actual reward. This prediction error is used to update expectations such that future prediction errors are minimized. A signal resembling this prediction error is coded by midbrain dopamine neurons.
Multiple-systems theory of decision-making: Machine learning research shows that there are different computational approaches to solving the problem of producing behavior that maximizes reward. Neural recordings suggest that each of these different algorithms are implemented in the brain, in distinct but overlapping areas.
Impulsivity: Impulsivity can refer to the inability to inhibit ongoing actions, inability to stick with a long-term plan, or unwillingness to make effort or wait to get a reward. Each of these phenomena reflects a lack of top-down or executive control. In this paper, we focus on unwillingness to wait for delayed rewards.
